# High expression of secretory leukocyte protease inhibitor (SLPI) in stage III micro-satellite stable colorectal cancer is associated with reduced disease recurrence

**DOI:** 10.1038/s41598-022-16427-5

**Published:** 2022-07-16

**Authors:** Sandrine Nugteren, Sjoerd H. den Uil, Pien M. Delis-van Diemen, Ytje Simons-Oosterhuis, Dicky J. Lindenbergh-Kortleve, Daniëlle H. van Haaften, Hein B. A. C. Stockmann, Joyce Sanders, Gerrit A. Meijer, Remond J. A. Fijneman, Janneke N. Samsom

**Affiliations:** 1 Division Gastroenterology and Nutrition, Laboratory of Pediatrics, Erasmus University Medical Center, Sophia Children’s Hospital, Room Ee1567A, P.O. Box 2040, 3000 CA Rotterdam, The Netherlands; 2 Department of Surgery, Spaarne Gasthuis, Haarlem, The Netherlands; 3 Department of Pathology, The Netherlands Cancer Institute, Amsterdam, The Netherlands

**Keywords:** Gastrointestinal cancer, Cancer, Immunology, Innate immunity, Mucosal immunology

## Abstract

Secretory leukocyte protease inhibitor (SLPI) is a pleiotropic protein produced by healthy intestinal epithelial cells. SLPI regulates NF-κB activation, inhibits neutrophil proteases and has broad antimicrobial activity. Recently, increased SLPI expression was found in various types of carcinomas and was suggested to increase their metastatic potential. Indeed, we demonstrated that SLPI protein expression in colorectal cancer (CRC) liver metastases and matched primary tumors is associated with worse outcome, suggesting that SLPI promotes metastasis in human CRC. However, whether SLPI plays a role in CRC before distant metastases have formed is unclear. Therefore, we examined whether SLPI expression is associated with prognosis in CRC patients with localized disease. Using a cohort of 226 stage II and 160 stage III CRC patients we demonstrate that high SLPI protein expression is associated with reduced disease recurrence in patients with stage III micro-satellite stable tumors treated with adjuvant chemotherapy, independently of established clinical risk factors (hazard rate ratio 0.54, *P*-value 0.03). SLPI protein expression was not associated with disease-free survival in stage II CRC patients. Our data suggest that the role of SLPI in CRC may be different depending on the stage of disease. In stage III CRC, SLPI expression may be unfavorable for tumors, whereas SLPI expression may be beneficial for tumors once distant metastases have established.

## Introduction

Secretory Leukocyte Protease Inhibitor (SLPI) is expressed and secreted mainly by human epithelial cells^1,2^. SLPI maintains intestinal homeostasis by preventing tissue destruction by neutrophil proteases and by regulating the threshold of inflammatory immune responses^3–5^. In the intestinal epithelium, SLPI expression is induced by repetitive microbial contact while it suppresses chemokine production by inhibiting nuclear factor kappa light chain enhancer of activated B cells (NF-κB) activation^5^.

Recently, increased SLPI protein expression has been demonstrated in various types of cancer, including colorectal cancer (CRC), gastric cancer, non-small cell lung cancer and ovarian cancer^6–9^. Interestingly, high tumor SLPI expression has been associated with poor prognosis. In particular, high tumor *SLPI* mRNA expression is associated with shorter overall survival in triple negative breast cancer patients^10^ and gastric cancer^7^. In addition, we previously showed that high SLPI protein expression in CRC liver metastases and matched primary tumors was associated with shorter overall survival^11^. The precise role of SLPI in cancer is, however, unclear. Clones expressing SLPI entered the vasculature more efficiently and formed more metastases than clones that did not express SLPI in a murine model of polyclonal breast cancer^12^. The role of SLPI in CRC metastasis has not extensively been studied. We previously hypothesized that SLPI expression may be beneficial for metastatic CRC tumor cells via promotion of immune evasion^11^. However, SLPI has multiple diverse functions and may act differently in various processes related to tumor growth and metastasis.

In this study, we assessed whether SLPI expression in CRC at a stage prior to distant metastases formation is associated with disease recurrence. We evaluated the prognostic value of SLPI protein expression in CRC patients classified as stage II (no metastases) and stage III (metastases to regional lymph nodes, but no distant metastases). Both stage II CRC patients and stage III CRC patients have a variable prognosis and therefore the identification of prognostic factors is desired to identify which patients may benefit from adjuvant therapy^13,14^. In addition, a better understanding of the biology of tumor growth and the formation of metastases may ultimately help to predict survival in CRC patients. Here, we show that high SLPI protein expression in micro-satellite stable (MSS) tumors is associated with reduced disease recurrence in stage III CRC patients treated with adjuvant chemotherapy.

## Methods

### Patient cohort and tissue microarray (TMA) generation

Between 1996 and 2005, 454 CRC patients classified as stage II or stage III according to the 4th edition of the TNM-classification system underwent surgical resection of CRC at the former Kennemer Gasthuis (current Spaarne Gasthuis) hospital in Haarlem, the Netherlands. Patients with a history of colorectal cancer, patients with irradical resection of the primary tumor, patients who died within 3 months after surgery and patients who were lost for follow-up were excluded from the study cohort (Supplementary Fig. 1). Histologically confirmed, formaldehyde-fixed paraffin-embedded (FFPE) CRC tissue samples from 386 patients were included, as described previously^15^. Tissue microarrays (TMAs) were generated from the original FFPE tissue blocks according to protocols previously described^16^. In short, six tissue core biopsies of 0.6 mm in diameter were punched from morphologically representative tissue areas and transferred into recipient TMA paraffin blocks. Tumor samples from this cohort have previously been analyzed for micro-satellite instability (MSI) using a five-marker-based PCR analysis system, as described previously^15^. For 48 out of 359 patients (13%) MSI status could not be determined. These patients were excluded from the analyses of SLPI expression in the subgroup with MSS tumors and the analyses of SLPI expression in the subgroup with MSI tumors.

### SLPI immunohistochemistry

Immunohistochemistry for SLPI was performed as previously described^11^. In short, sections were stained with either a monoclonal anti-human-SLPI antibody that was raised against human SLPI purified from sputum (4 μg/mL, mouse IgG1, HM2037, clone 31; HycultBiotech, Uden, The Netherlands) or a polyclonal anti-human-SLPI antibody that was raised against *Escherichia coli*-derived recombinant human SLPI (1 μg/mL, goat IgG, BAF1274; R&D Systems/Bio-Techne, Minneapolis, MN, USA). Sections were counterstained with hematoxylin (Vector Laboratories).

Previously, staining with the monoclonal antibody resulted in a clear signal with limited background staining^11^. Staining with the polyclonal antibody showed more background staining, but confirmed the staining patterns observed with the monoclonal antibody^11^. Therefore, in this study we focused on the results obtained with the monoclonal antibody and we used the polyclonal antibody again to support our findings.

### Scoring of SLPI expression

Images of stained sections were digitally captured using an Aperio AT2 scanner (Leica Microsystems B.V., Amsterdam, The Netherlands) equipped with a 20 × /0.75 objective (UPlanSAPO; Olympus, Leiderdorp, The Netherlands). SLPI intensity in the cytoplasm of neoplastic epithelial cells was manually scored in a semi-quantitative manner as ‘negative’, ‘weak’, ‘moderate’ or ‘strong’ using the online platform Slide Score (www.slidescore.com). The scoring strategy was designed based on the range of SLPI staining intensity observed among all tissue cores and was agreed on in consultation with a pathologist. All sections were scored by the same investigator. In order to assess the reproducibility of the scoring, a second pathologist independently scored > 25% of the cores with the monoclonal antibody and > 25% of the cores stained with the polyclonal antibody. Both observers were blinded to the clinical information at time of assessment. The linear weighted kappa values were 0.49 for the monoclonal antibody and 0.59 for the polyclonal antibody, indicating moderate interobserver agreement.

### Statistical analysis

For both anti-SLPI antibodies, the maximum score from the one to six TMA cores stained for each patient was used in all analyses. Patients for whom none of the six cores were evaluable were excluded from the analysis (Supplementary Fig. 1). Disease recurrence was defined as either local tumor recurrence or distant metastases or both, as diagnosed by computed tomography or histopathology. In order to assess the prognostic value of SLPI in stage II and stage III CRC, we determined the optimal cut-offs for dichotomization of the cohort by SLPI protein expression based on 5-years disease-free survival. First, the study population was randomly divided into five subsets. The optimal cut-off for dichotomizing four-fifth of the study population was calculated using receiver operating characteristic (ROC) curve analysis for 5-year disease-free survival. This procedure was repeated five times with one-fifth of the data set varying. The cut-off most often selected was chosen as the optimal cut-off. Patients were classified as ‘SLPI-low’ or ‘SLPI-high’ based on this validated cut-off for both antibodies separately. For CRC stained with the monoclonal antibody, the optimal cut-off was negative vs weak/moderate/strong SLPI expression. For CRC stained with the polyclonal antibody, the optimal cut-off was negative/weak vs moderate/strong SLPI expression. The validated cut-offs were subsequently applied to the study population to calculate a hazard rate ratio (HRR) and 95% confidence interval for 5-year disease-free survival in univariable Cox regression analysis. In addition, a corrected HRR was calculated by multivariable Cox regression analysis for 5-year disease-free survival. The relation between SLPI expression and disease-free survival was visualized by Kaplan Meier curves. The log-rank test was used to determine whether disease-free survival varied significantly between the SLPI-low and SLPI-high group. Categorical clinicopathological features of the SLPI-low and SLPI-high groups were compared using Pearson’s Chi-squared test or Fishers exact test (two-sided) in case of expected frequencies < 5. Differences between the SLPI-low and SLPI-high groups in clinicopathological features that were measured on a continuous scale and not normally distributed were compared using the Kruskal–Wallis rank sum test. The relationship between SLPI detected using the monoclonal antibody and SLPI detected using the polyclonal antibody was examined using Fisher’s exact test (two-sided). All statistical analyses and visualization were performed using R version 3.5.1^17^. The ‘survival’^18,19^, ‘survminer’^20^, ‘pROC’^21^ and ‘survivalROC’^22^ packages were employed for survival analysis and to calculate the optimal cut-offs. All methods were carried out in accordance with the REMARK recommendations for reporting tumor marker prognostic studies^23^.

### Ethics approval and consent to participate

Collection, storage and use of the tissue samples and clinical data of the cohort were conducted in compliance with the Dutch code of conduct for responsible use of human tissue for medical research^24^. Use of this cohort for the present study was evaluated and approved by the Institutional Review Board of the Netherlands Cancer Institute (IRBd18159). Collection of patient data and tissue samples was according to local and national legislation at the time of sample and data collection and in compliance with the ‘Code for Proper Secondary Use of Human Tissue in The Netherlands’. This allowed the present retrospective observational study to be performed without the need for study-specific informed consent from individual patients. Experimental protocols needed for the current study were approved and the need for informed consent was waived by The Netherlands Cancer Institute, Amsterdam, The Netherlands.

## Results

### SLPI is expressed in a subset of stage II and stage III CRC

To establish the prognostic value of SLPI in stage II and stage III CRC we assessed SLPI protein expression in CRC tissue samples from a Dutch cohort of 226 stage II and 160 stage III CRC patients. The characteristics of this study population have been described previously^15^. CRC tissue samples from in total 359 patients were available for analysis of SLPI stained with the monoclonal antibody (Supplementary Fig. 1). We focused on the results obtained with the monoclonal antibody.

**Figure 1:**
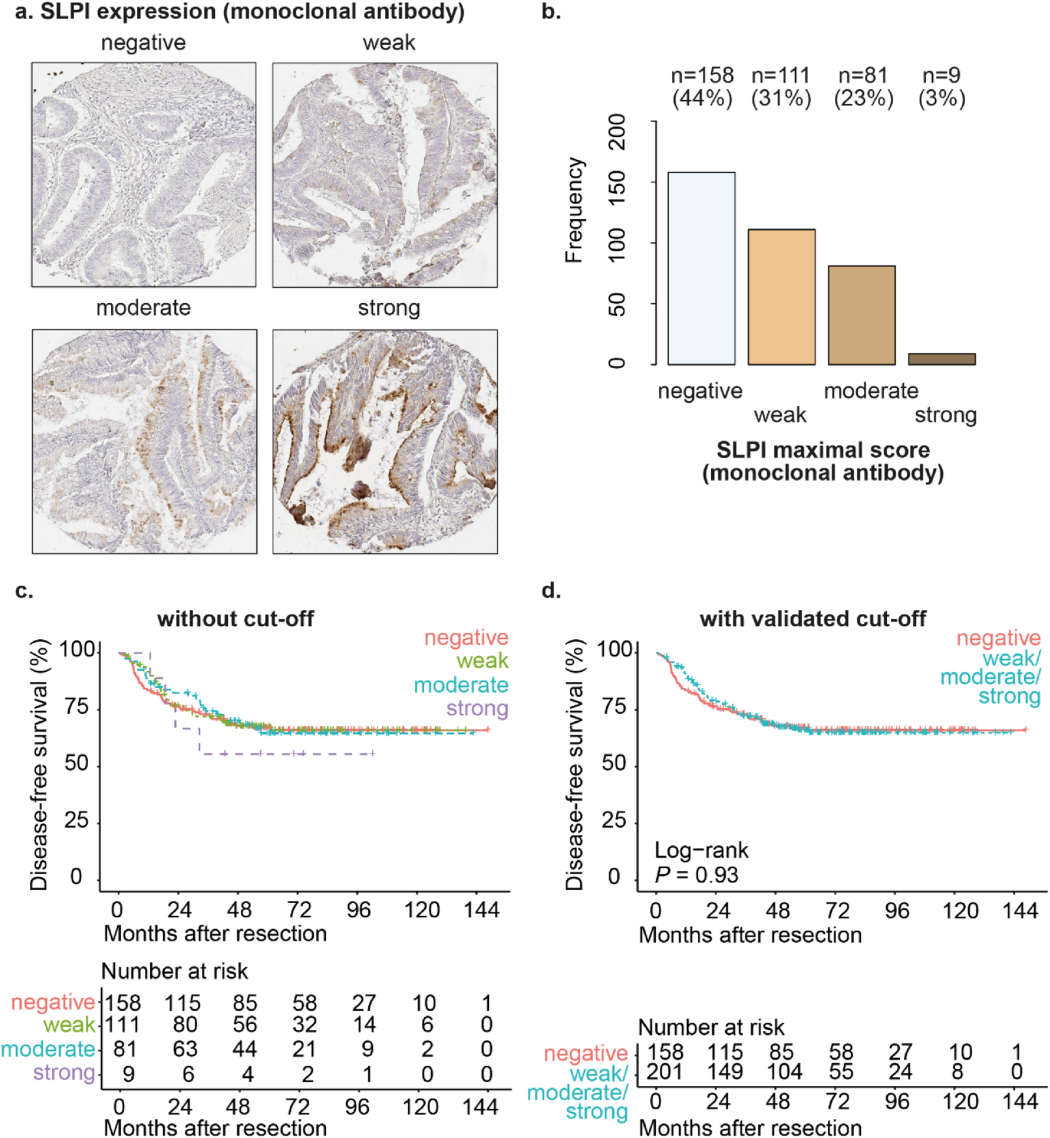
SLPI is expressed in a subset of stage II and stage III CRC. Examples of TMA cores of stage II or stage III CRC stained for SLPI using the monoclonal antibody (**a**). Frequencies and percentages of stage II or stage III CRC scored as ‘negative’, ‘weak’, ‘moderate’ or ‘strong’ after staining with the monoclonal SLPI antibody (**b**); only the maximum score for each patient was included. Kaplan–Meier curves for disease-free survival after resection of the primary tumor (in months) for the total study population of stage II and stage III CRC patients stratified by SLPI expression detected using the monoclonal antibody (**c**,**d**). Curves without a cut-off (**c**) and with the validated cut-off (**d**) are shown. *P*-values were calculated using the log-rank test.

We observed SLPI expression in the cytoplasm of the tumor cells; in some tissue cores mainly on the luminal side of the tumor cells and in other tissue cores in the whole cytoplasm of the tumor cells (Fig. 1a and Supplementary Fig. 4a). We detected expression of SLPI in CRC in 56% of patients (Fig. 1b). Using the validated cut-offs, SLPI expression was not significantly different between stage II and stage III CRC patients (Supplementary Fig. 2). In conclusion, we detected SLPI protein expression in a substantial subgroup of stage II and stage III CRC patients.

### SLPI expression in the whole cohort of stage II CRC or stage III CRC is not associated with disease-free survival

When evaluating stage II and stage III CRC patients together, SLPI expression was not associated with disease-free survival (HRR 0.98, *P*-value 0.93, 95% confidence interval 0.68–1.42, Fig. 1c,d). Patient age, gender, grade of differentiation of the tumor, tumor stage, nodal stage, presence of mucinous differentiation, presence of micro-satellite instability (MSI), presence of ulceration, presence of angio-invasion, occurrence of perforation, occurrence of tumor spill, treatment with adjuvant chemotherapy, disease recurrence (local or distant), CRC-related mortality, overall mortality and the follow-up time were not significantly different between patients with high or low SLPI expression in CRC tissues (Supplementary Fig. 2a). Patients with high SLPI expression significantly more often had left-sided tumors and significantly smaller tumors (Supplementary Fig. 2a).

**Figure 2:**
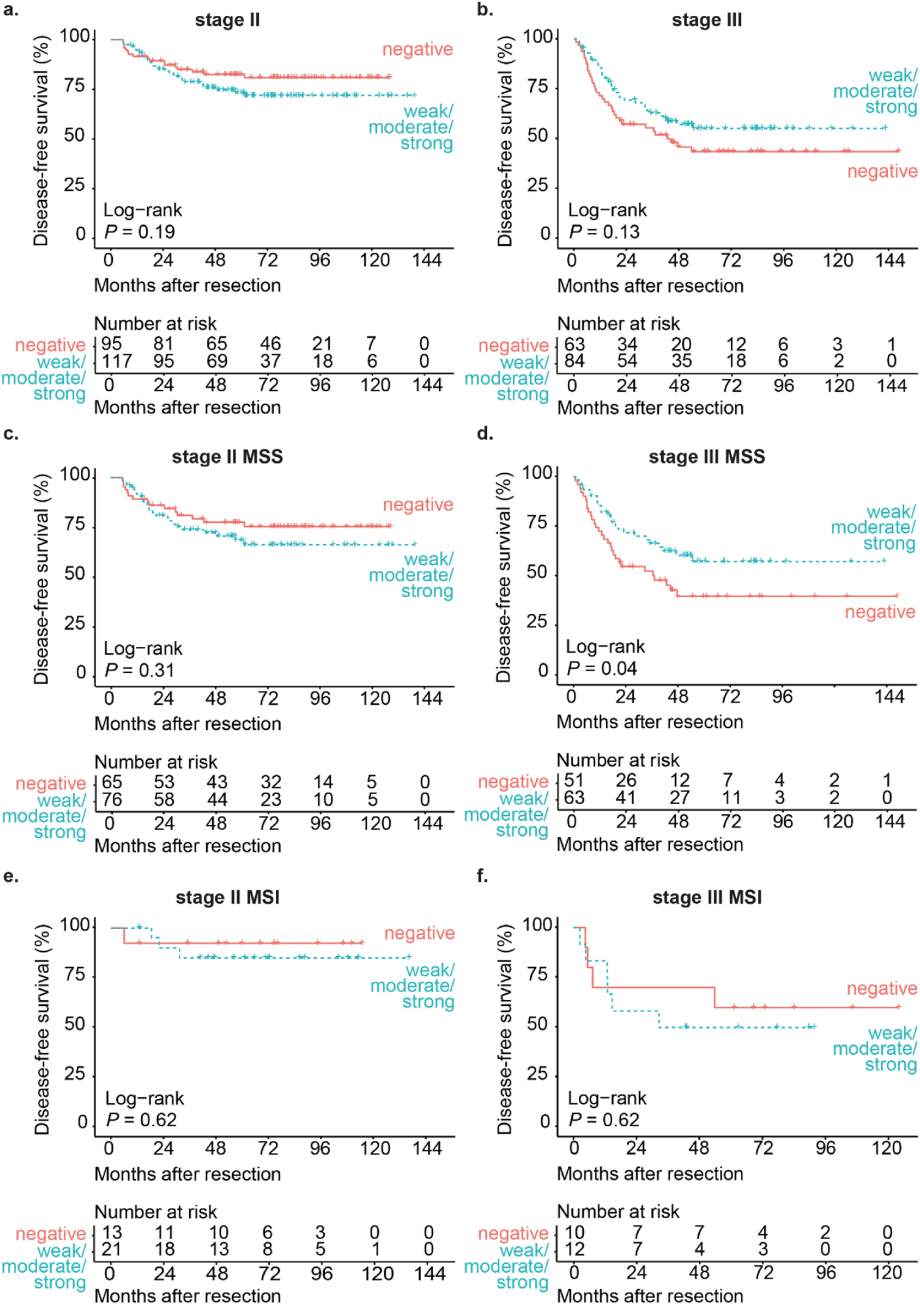
High SLPI expression in stage III MSS CRC is associated with increased disease-free survival. Kaplan–Meier curves for disease-free survival after resection of the primary tumor (in months) for either stage II CRC patients (**a**) or stage III CRC patients (**b**) stratified by SLPI expression detected using the monoclonal antibody. Kaplan–Meier curves for disease-free survival after resection of the primary tumor (in months) for stage II CRC patients with MSS (**c**) or MSI tumors (**e**) and stage III CRC patients with MSS (**d**) or MSI tumors (**f**) stratified by SLPI expression detected using the monoclonal antibody. Curves with the validated cut-off are shown. *P*-values were calculated using the log-rank test. *MSS* micro-satellite stable, *MSI* micro-satellite instable.

When evaluating stage II CRC patients separately, SLPI expression was not associated with disease-free survival (HRR 1.48, *P*-value 0.19, 95% confidence interval 0.81–2.69, Fig. 2a). When evaluating stage III CRC patients separately, SLPI expression was also not associated with disease-free survival (HRR 0.70, *P*-value 0.13, 95% confidence interval 0.43–1.12, Fig. 2b). There were no significant differences in clinicopathological characteristics in stage II or stage III patients between the low-SLPI and the high-SLPI group (Supplementary Fig. 2b and 2c).

### High expression of SLPI in stage III micro-satellite stable CRC is associated with reduced disease recurrence

Because patients with micro-satellite instable (MSI) CRC are known to have a better prognosis compared to patients with micro-satellite stable (MSS) CRC^25–27^, we evaluated the prognostic value of SLPI in the subgroup of patients with MSS tumors and in the subgroup of patients with MSI tumors separately. In stage II patients with MSS tumors, SLPI expression was not associated with disease-free survival (HRR 1.40, *P*-value 0.31, 95% confidence interval 0.73–2.68, Fig. 2c). In stage II patients with MSI tumors, SLPI expression was also not associated with disease-free survival (HRR 1.77, *P*-value 0.62, 95% confidence interval 0.18–17.01, Fig. 2e).

In contrast, in stage III patients with MSS tumors high SLPI expression was associated with a significantly increased disease-free survival (HRR 0.58, *P*-value 0.04, 95% confidence interval 0.34–0.99, Fig. 2d). Mucinous differentiation was significantly more often present in stage III patients with MSS tumors in the SLPI-high group compared to the SLPI-low group (*P*-value < 0.01, Supplementary Fig. 2d). Mucinous differentiation was not associated with disease-free survival in this subgroup (HRR 0.99, *P*-value 0.98, 95% confidence interval 0.49–2.02) and is therefore not likely to confound the association between SLPI and disease-free survival in stage III patients with MSS tumors. In stage III CRC patients with MSI tumors, SLPI expression was not associated with disease-free survival (HRR 1.38, *P*-value 0.62, 95% confidence interval 0.39–4.93, Fig. 2f). In conclusion, high SLPI expression is associated with reduced disease recurrence in stage III CRC patients with MSS tumors.

### High expression of SLPI in micro-satellite stable CRC is associated with reduced disease recurrence in stage III patients treated with adjuvant chemotherapy

**Figure 3:**
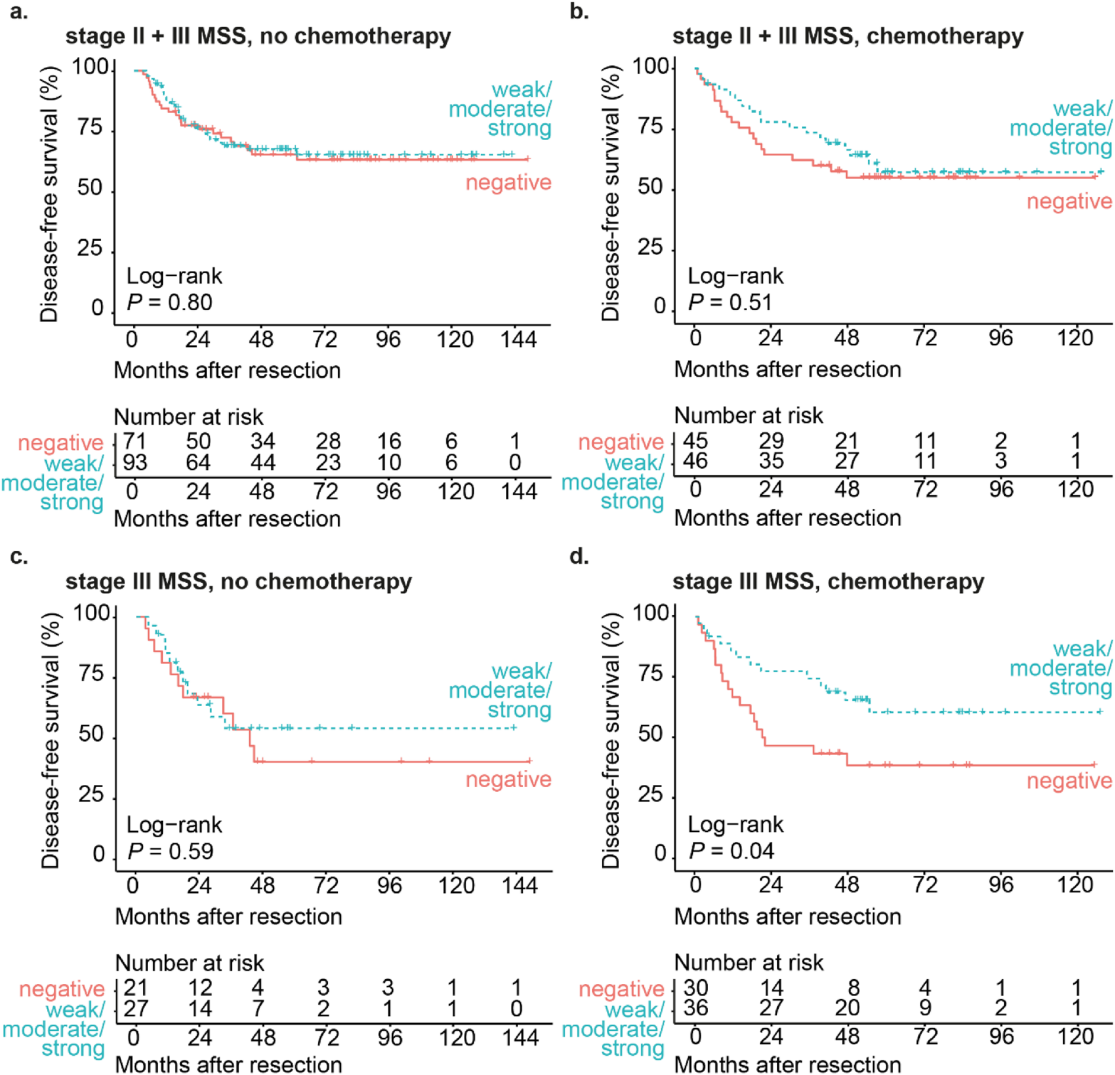
High SLPI expression in chemotherapy-treated stage III MSS CRC patients is associated with increased disease-free survival. Kaplan–Meier curves for disease-free survival after resection of the primary tumor (in months) for stage II and stage III CRC patients with MSS tumors not treated with adjuvant chemotherapy (**a**) or treated with adjuvant chemotherapy (**b**) stratified by SLPI expression detected using the monoclonal antibody. Kaplan–Meier curves for disease-free survival after resection of the primary tumor (in months) for stage III CRC patients with MSS tumors not treated with adjuvant chemotherapy (**c**) or treated with adjuvant chemotherapy (**d**) stratified by SLPI expression detected using the monoclonal antibody. Curves with the validated cut-off are shown. *P*-values were calculated using the log-rank test. *MSS* micro-satellite stable.

As a substantial group of stage III CRC patients in this cohort has been treated with 5FU-based adjuvant chemotherapy, which is likely to affect disease-free survival, we evaluated the association between SLPI and disease-free survival in patients with MSS tumors separately for patients who did and who did not receive adjuvant chemotherapy. When we stratified for adjuvant chemotherapy in the whole cohort of stage II and stage III MSS CRC, SLPI expression was not associated with disease-free survival in patients who did not receive adjuvant chemotherapy (HRR 0.93, *P*-value 0.80, 95% confidence interval 0.54–1.60, Fig. 3a) or in patients who received adjuvant chemotherapy (HRR 0.81, *P*-value 0.51, 95% confidence interval 0.43–1.52, Fig. 3b).

In stage III patients with MSS tumors who did not receive adjuvant chemotherapy, SLPI expression was not significantly associated with disease-free survival (HRR 0.80, *P*-value 0.59, 95% confidence interval 0.34–1.84, Fig. 3c). To determine whether SLPI has prognostic value in stage III MSS CRC patients who received adjuvant chemotherapy, we assessed disease free survival in patients with low SLPI expression and high SLPI expression in the 58% of stage III MSS CRC patients who received adjuvant chemotherapy (Fig. 3c,d). In stage III MSS patients who received adjuvant chemotherapy, high SLPI expression was significantly associated with increased disease-free survival (HRR 0.48, *P*-value 0.04, 95% confidence interval 0.23–0.98, Fig. 3d). In these patients, clinicopathological characteristics were not different between the low-SLPI and the high-SLPI group (Supplementary Fig. 2e). These findings raised the question whether treatment with adjuvant chemotherapy could explain the association between high SLPI expression and reduced disease recurrence in stage III MSS CRC patient. However, treatment with adjuvant chemotherapy was not significantly associated with disease recurrence in stage III MSS colorectal cancer patients (HRR 1.04, P-value 0.89, 95% confidence interval 0.61–1.78) and the proportion of patients who received adjuvant chemotherapy was not different between the low-SLPI and the high-SLPI group (Supplementary Fig. 2d). Therefore, the association between high SLPI expression in stage III MSS colorectal cancer and reduced disease recurrence cannot be explained by treatment with adjuvant chemotherapy.

In conclusion, high SLPI expression is associated with reduced disease recurrence in stage III patients with MSS tumors who did receive adjuvant chemotherapy, but not in stage III patients with MSS tumors who did not receive adjuvant chemotherapy.

### SLPI expression has prognostic value in stage III patients with MSS CRC independently of established clinical risk factors

Next, we investigated whether the prognostic value of SLPI expression in stage III CRC patients with MSS tumors was independent of previously established clinical risk factors. The following factors have been previously demonstrated to be associated with prognosis in CRC patients: tumor location, tumor stage, nodal stage, isolated tumor deposits, angio-invasion, tumor histological grade, ulceration, perforation and tumor spill^28–30^.

In stage III patients with MSS tumors, the following factors were associated with disease recurrence in the univariable Cox regression with a *P*-value of 0.1 or below and were therefore included in the multivariable Cox regression model: tumor stage (HRR 1.86, *P*-value 0.01, 95% confidence interval 1.16–3.00), nodal stage (HRR 1.57, *P*-value 0.1, 95% confidence interval 0.91–2.71), isolated tumor deposits (HRR 1.86, *P*-value 0.03, 95% confidence interval 1.05–3.29), angio-invasion (HRR 2.65, *P*-value < 0.01, 95% confidence interval 1.56–4.49), tumor histological grade (HRR 1.80, *P*-value 0.05, 95% confidence interval 1.00–3.25) and perforation (HRR 2.21, *P*-value 0.03, 95% confidence interval 1.04–4.68). The prognostic value of SLPI expression was not confounded by these factors as high SLPI expression was associated with reduced disease recurrence in the multivariable model (HRR 0.54, *P-*value 0.03, 95% confidence interval 0.31–0.94). Factors that were significantly associated with disease recurrence in this model were angio-invasion (HRR 2.88, *P*-value < 0.01, 95% confidence interval 1.53–5.42) and perforation (HRR 3.04, *P*-value < 0.01, 95% confidence interval 1.38–6.70). In conclusion, high SLPI expression in MSS tumors of stage III CRC patients was associated with significantly reduced disease recurrence after surgical resection of the primary tumor, independently of known clinical risk factors.

In addition, we evaluated whether SLPI expression also had prognostic value independently of clinical risk factors in stage III CRC patients with MSS tumors who received adjuvant chemotherapy, as in these patients high SLPI expression was associated with reduced disease recurrence. In this subgroup, the following clinical risk factors were associated with disease recurrence in the univariable Cox regression with a *P*-value of 0.1 or below and were therefore included in the multivariable Cox regression model: tumor stage (HRR 1.96, *P*-value 0.03, 95% confidence interval 1.08–3.57), nodal stage (HRR 1.77, *P*-value 0.1, 95% confidence interval 0.90–3.48), isolated tumor deposits (HRR 1.96, *P*-value 0.06, 95% confidence interval 0.97–3.97), angio-invasion (HRR 3.51, *P*-value < 0.01, 95% confidence interval 1.76–7.00), tumor histological grade (HRR 1.74, *P*-value 0.1, 95% confidence interval 0.82–3.70), ulceration (HRR 0.40, *P*-value 0.02, 95% confidence interval 0.18–0.86), perforation (HRR 2.84, *P*-value 0.07, 95% confidence interval 0.87–9.34) and tumor spill (HRR 14.18, *P*-value < 0.01, 95% confidence interval 2.73–73.6). The prognostic value of SLPI expression was not confounded by these factors (HRR 0.43, *P-*value 0.03, 95% confidence interval 0.20–0.93). The factors that remained significantly associated with disease recurrence in this model were angio-invasion (HRR 3.13, *P*-value 0.01, 95% confidence interval 1.29–7.61) and perforation (HRR 4.49, *P*-value 0.04, 95% confidence interval 1.11–18.13). In conclusion, detection of high SLPI expression in MSS tumors of stage III CRC patients who received adjuvant chemotherapy was associated with significantly reduced disease recurrence after surgical resection the primary tumor, independently of known clinical risk factors.

### Detection of SLPI with the polyclonal antibody supports the association between high expression of SLPI in stage III micro-satellite stable CRC and reduced disease recurrence

To robustly assess the prognostic value of SLPI, we detected SLPI protein expression by immunohistochemistry using both a monoclonal antibody and a polyclonal antibody. Previously, we observed a significant association between SLPI detected with the monoclonal antibody and SLPI detected with the polyclonal antibody^11^. The monoclonal antibody had better discriminative potential and revealed a stronger association between SLPI and prognosis in patients with CRC liver metastases, and results obtained with the polyclonal antibody confirmed our findings^11^. Therefore, we also assessed in the current study whether SLPI staining with the polyclonal antibody revealed the association between high expression of SLPI in MSS tumors and reduced disease recurrence in stage III CRC patients.

Using the polyclonal antibody, we detected expression of SLPI in the cytoplasm of tumor cells of 96% of CRC patients (Supplementary Fig. 4a and 4b). We compared the scores for SLPI expression stained with the monoclonal antibody with the scores for SLPI expression stained with the polyclonal antibody in 349 patients (Supplementary Fig. 3). There was a significant association between detection of high SLPI expression with the monoclonal antibody and detection of high SLPI expression with the polyclonal antibody (two-sided Fisher’s exact test: *P-*value < 0.01). Both staining with the monoclonal and polyclonal antibody resulted in the same classification as either SLPI-low or SLPI-high for 77% of patients, based on the validated cut-offs (Supplementary Fig. 3). SLPI expression scores were more frequently lower in CRC stained with the monoclonal antibody compared to the polyclonal antibody than vice versa, which indicates that the monoclonal antibody has a higher threshold of detection for SLPI, as we observed previously^11^.

Detection of SLPI using the polyclonal antibody led to the same trends as the data obtained with the monoclonal antibody. SLPI expression detected with the polyclonal antibody was not associated with disease-free survival in the whole cohort of stage II and stage III CRC patients (HRR 0.91, *P*-value 0.60, 95% confidence interval 0.63–1.31, Supplementary Fig. 4c and 4d) or in stage II patients separately (HRR 1.16, *P*-value 0.61, 95% confidence interval 0.65–2.08, Supplementary Fig. 5a). In stage III CRC patients, we observed a non-significant trend between high SLPI expression and increased disease-free survival (HRR 0.70, *P*-value 0.14, 95% confidence interval 0.44–1.13, Supplementary Fig. 5b). In stage III patients with MSS tumors, we also observed a trend between high SLPI expression detected with the polyclonal antibody and increased disease-free survival (HRR 0.62, *P*-value 0.08, 95% confidence interval 0.36–1.06, Supplementary Fig. 5d). Distant disease recurrence was significantly more frequent in the SLPI-low group when SLPI was detected with the polyclonal antibody (Supplementary Fig. 7d), which fits with the association between high SLPI expression and increased disease-free survival. We observed similar results in stage III patients with MSS tumors who received adjuvant chemotherapy (Supplementary Fig. 6d and Supplementary Fig. 7e).

## Discussion

SLPI is expressed by healthy intestinal epithelial cells throughout the body and maintains tissue homeostasis by preventing protease-induced tissue damage and by regulating inflammatory immune responses. We previously demonstrated that high SLPI expression in CRC liver metastases and matched primary tumors of patients with colorectal liver metastases is associated with poorer overall survival^11^. However, the prognostic value of SLPI in CRC patients with localized disease has not been established. Here, we studied SLPI protein expression in a large cohort of stage II and stage III CRC patients and found that high SLPI protein expression in MSS tumors is associated with reduced disease recurrence after resection of the primary tumor in stage III CRC patients. In particular, high SLPI expression was associated with reduced disease recurrence in stage III MSS CRC patients treated with 5FU-based adjuvant chemotherapy, independently of established clinical risk factors.

Previously, we observed that high SLPI expression in liver metastases and matched primary tumors is associated with worse prognosis^11^. The data presented in the current study may appear contradictive, as we find an association between high SLPI expression and reduced disease recurrence in stage III CRC patients, while recurrence most often involves distant metastasis formation. In addition, we do not find an association between SLPI expression and disease recurrence in stage II CRC patients. Our data therefore argue that regulation of SLPI expression or its function in CRC may vary depending on the stage of the disease. In the current study, primary tumor tissue was obtained from patients with lymph node metastases prior to formation of distant metastases. At this stage, SLPI expression in the primary tumor appears to reflect a biological process which is unfavorable to the tumor and is associated with reduced formation of distant metastases at a later time. In contrast, in the previous cohort all patients developed liver metastases and in a substantial number of patients liver metastases were found at the same time or within 6 months after detection of the primary tumor^31^. The primary tumors of patients in the liver metastases cohort are therefore likely at a more advanced stage compared to the primary tumors of stage III patients in the current study. In addition, in the previous study we assessed overall survival after resection of liver metastases, whereas in the current study the outcome was defined as either local disease recurrence, development of distant metastases or both. It would be interesting to investigate whether SLPI expression in the current cohort is associated with poorer survival after the disease has recurred, but the subgroup of patients with disease recurrence was too low to have sufficient power to assess this. Thus, we studied different steps in the progression of CRC in this cohort and our previous cohort and we show that SLPI expression may relate to different biological processes or have a different consequence for the tumor.

There are multiple possible explanations for our findings. Firstly, tumor cells expressing SLPI in stage III patients may be different from the tumor cells which later form metastases and were studied in our CRC liver metastases cohort. In differentiated tumor cells SLPI expression may reflect a response to the tumor microenvironment, whereas in more aberrant cells SLPI expression may reflect increased metastatic potential. Unfortunately, based on our current analyses we cannot assess whether SLPI-expressing tumor cells from stage III patients are indeed different from those in the primary tumors of patients with liver metastases. Of note, we did observe that SLPI expression in MSS tumors from stage III patients is associated with mucinous differentiation, but in the CRC liver metastases cohort mucinous differentiation was not assessed.

Alternatively, irrespective of whether there is a difference in the tumor cells expressing SLPI, SLPI may exert opposing functions during the course of disease. Although stage III CRC patients have lymph node metastases, tumor cells need to acquire different functionalities for the formation of distant metastases and therefore the role of SLPI in this stage could be different. For example, during distant metastasis formation SLPI can induce the formation of vessel-like structures which provide a blood supply to hypoxic regions of the tumor^12^. Moreover, SLPI acts as an anticoagulant^12^ and inhibits the antiangiogenic factor Endostatin^32^. SLPI may also promote vascular invasion via induction of MMP-2 and MMP-9 production by tumor cells^10^. These tumor cell functions may not yet be active in primary tumor cells in stage III CRC patients. In contrast, in the stage prior to distant metastasis formation SLPI expression in the tumor may counteract other tumor cell functions. For example, SLPI can inhibit tumor growth, suppress migration and promote apoptosis in vitro^33–36^. However, the precise role of SLPI in the different steps of CRC metastasis formation remains to be investigated.

High SLPI expression was only associated with reduced disease recurrence in stage III CRC patients with MSS tumors, in contrast to patients with MSI tumors. The reason for this specific association is unclear. MSI tumors are known to elicit strong anti-tumor immune responses relative to MSS tumors^37^. SLPI expression can be modulated by inflammation and ensuing toll-like receptor signaling. As such, it could have been expected that SLPI expression is higher in MSI tumors and also impacts disease recurrence in this group of patients. However, MSI status was not associated with the degree of SLPI expression in tumors of stage II or stage III patients. Possibly, inhibition of NF-κB by SLPI is more relevant in MSS tumors compared to MSI tumors, as dedifferentiation of tumor cells due to NF-κB-induced Wnt-signaling may particularly occur in MSS CRC^38^.

We only observed the association between high SLPI expression in MSS tumors and reduced disease recurrence in stage III patients who did receive adjuvant chemotherapy (Fig. 3d) and not in patients who did not receive adjuvant chemotherapy (Fig. 3c). This suggests that high SLPI expression may predispose to better killing of tumor cells by 5FU-based adjuvant chemotherapy. Possibly, the formation of vessel-like structures and anticoagulation induced by SLPI^12^ make hypoxic regions of the tumor more accessible to chemotherapy.

## Conclusion

In conclusion, high SLPI expression in MSS CRC is associated with reduced disease recurrence after resection of the primary tumor and adjuvant chemotherapy in stage III patients. In view of the association between high SLPI expression in CRC liver metastases and matched primary tumors and poorer overall survival, these data suggest different roles for SLPI in CRC before and after the formation of distant metastases. In addition, high SLPI expression in MSS tumors may predict a better response to adjuvant chemotherapy in stage III CRC patients.

## Supplementary Information

Supplementary Information 1.

Supplementary Information 2.

Supplementary Information 3.

## Data Availability

The dataset supporting the conclusions of this article is included within the article (Supplementary Dataset File 1).

## Abbreviations

SLPI: Secretory leukocyte protease inhibitor
CRC: Colorectal cancer
NF-κB: Nuclear factor kappa light chain enhancer of activated B cells
TMA: Tissue microarray
MSS: Micro-satellite stable
MSI: Micro-satellite instability
ROC: Receiver operating characteristic
HRR: Hazard rate ratio
