# Supplementary material for: High expression of secretory leukocyte protease inhibitor (SLPI) in stage III micro-satellite stable colorectal cancer is associated with reduced disease recurrence
Source: Sci Rep. 2022 Jul 16;12:12174. doi: 10.1038/s41598-022-16427-5 (PMC9288430; doi:10.1038/s41598-022-16427-5)
Supplement: Supplementary file 1 — Supplementary Information 1. [file 41598_2022_16427_MOESM1_ESM.pdf]

Supplementary Dataset File 1: Data used to generate Kaplan-Meier curves. Anonymous patient number; dichotomized scores for SLPI stained with the monoclonal antibody or polyclonal antibody (0 = ‘SLPI-low’ and 1 = ‘SLPI-high’ based on the validated cut-offs); disease-free survival after resection of the primary tumor (in months); whether the patient developed a relapse or not; microsatellite instability status of the primary tumor (MSS = microsatellite stable, MSI = microsatellite instable); disease stage; and whether the patient received adjuvant chemotherapy or not.
