# Supplementary material for: High expression of secretory leukocyte protease inhibitor (SLPI) in stage III micro-satellite stable colorectal cancer is associated with reduced disease recurrence
Source: Sci Rep. 2022 Jul 16;12:12174. doi: 10.1038/s41598-022-16427-5 (PMC9288430; doi:10.1038/s41598-022-16427-5)
Supplement: Supplementary file 3 — Supplementary Information 3. [file 41598_2022_16427_MOESM3_ESM.docx]

**Supplementary figure 1:**

**
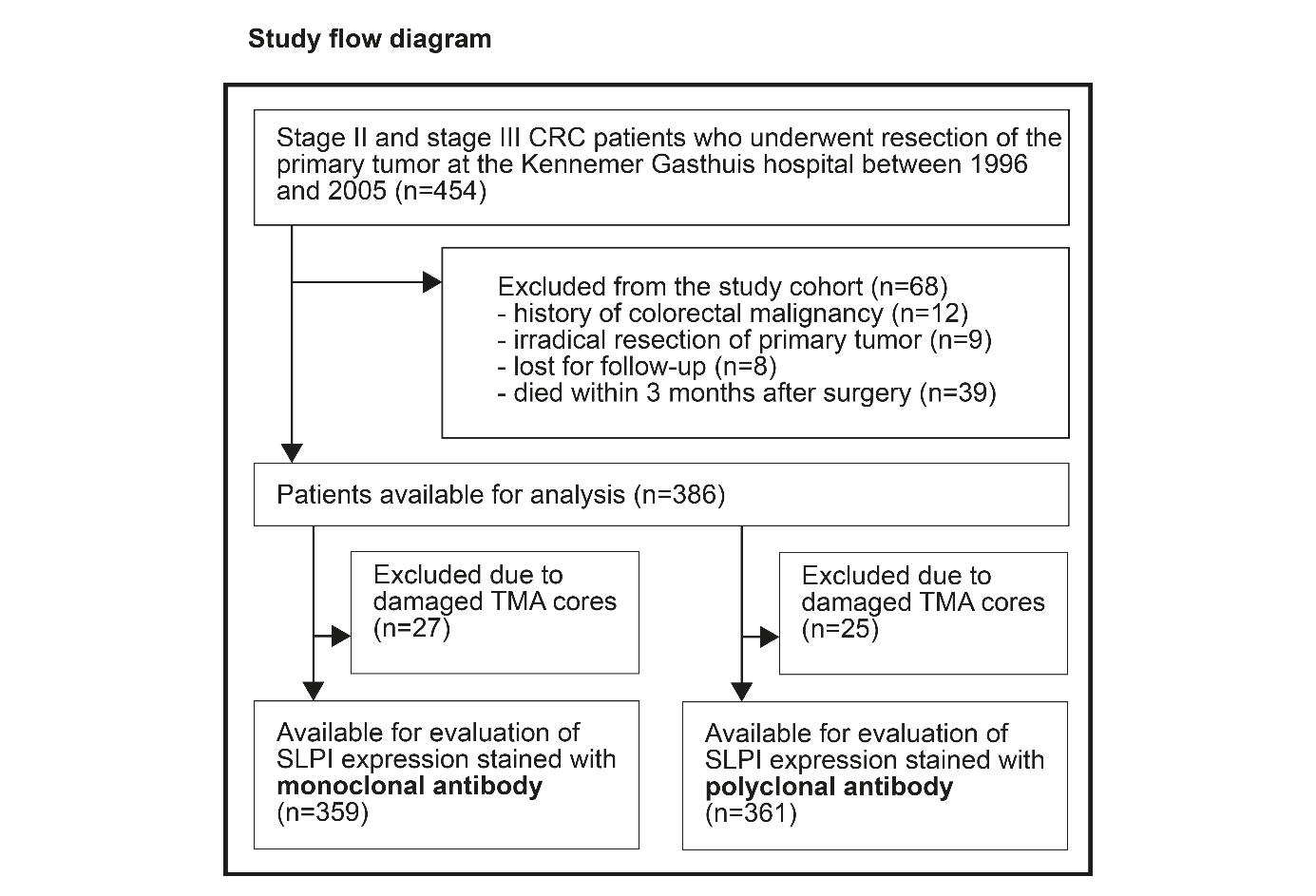
**

**Supplementary figure 1: Flow diagram of the study cohort.** In total, 454 patients with stage II or stage III CRC underwent resection of the primary tumor at the Kennemer Gasthuis hospital between 1996 and 2005. Patients with a history of colorectal cancer, patients with an irradical resection of the primary tumor, patients who died within 3 months after the resection and patients who were lost for follow-up were excluded, resulting in a study cohort of 386 patients. After excluding patients for whom none of the six cores could be scored due to technical reasons, SLPI expression was scored in CRC stained with the monoclonal antibody from 359 patients and CRC stained with the polyclonal antibody from 361 patients.

**Supplementary figure 2:**

**a. Characteristics of stage II and stage III patients included in the analysis of SLPI expression using the monoclonal antibody**

| **Clinicopathological variable** | **SLPI-low (n=158)** | **SLPI-high (n=201)** | **Total (n=359)** | ***P*-value** |
| --- | --- | --- | --- | --- |
| Age in years, median (min - max) | 71  (28 – 92) | 73  (35 – 92) | 72  (28 – 92) | 0.26^1^ |
| Gender  female  male | 70 (44%)  88 (56%) | 99 (49%)  102 (52%) | 169 (47%)  190 (53%) | 0.35^2^ |
| Tumor location  right (cecum until flexura lienalis)  left (flexura lienalis to rectum) | 79 (50%)  79 (50%) | 79 (39%)  122 (61%) | 158 (44%)  201 (56%) | **0.04^2^** |
| Tumor histological grade  well-differentiated  moderately differentiated  poorly differentiated | 8 (5%)  199 (75%)  31 (20%) | 12 (6%)  164 (82%)  25 (12%) | 20 (6%)  283 (79%)  56 (16%) | 0.17^2^ |
| Tumor maximal diameter in mm, median  (min - max) | 40  (15 – 130) | 35  (10 – 100) | 40  (10 – 130) | **0.03^1^** |
| Stage  II III | 95 (60%)  63 (40%) | 117 (58%)  84 (42%) | 212 (59%)  147 (41%) | 0.71^2^ |
| Tumor stage  T1  T2  T3  T4 | 2 (1%)  5 (3%)  134 (84%)  17 (11%) | 2 (1%)  14 (7%)  169 (84%)  16 (8%) | 4 (1%)  19 (5%)  303 (84%)  33 (9%) | 0.34^3^ |
| Nodal stage (Stage III patients only)  N1  N2 | 40 (64%)  23 (37%) | 60 (71%)  24 (29%) | 100 (68%)  47 (32%) | 0.31^2^ |
| Mucinous differentiation | 27 (17%) | 48 (24%) | 75 (21%) | 0.12^2^ |
| MSI-status  MSI  MSS  Unknown | 23 (15%)  116 (73%)  19 (12%) | 33 (16%)  139 (69%)  29 (14%) | 56 (16%)  255 (71%)  48 (13%) | 0.55^2^ |
| Ulceration | 125 (79%) | 152 (76%) | 277 (77%) | 0.43^2^ |
| Angio-invasion | 34 (22%) | 36 (18%) | 70 (20%) | 0.39^2^ |
| Emergency surgery | 21 (13%) | 26 (13%) | 47 (13%) | 0.92^2^ |
| Perforation (pre-/per-/post-operative) | 12 (8%) | 19 (10%) | 31 (9%) | 0.53^2^ |
| Tumor spill | 4 (3%) | 8 (4%) | 12 (3%) | 0.45^2^ |
| Adjuvant chemotherapy | 52 (33%) | 64 (32%) | 116 (32%) | 0.83^2^ |
| Disease recurrence | 51 (32%) | 65 (32%) | 116 (32%) | 0.99^2^ |
| Local disease recurrence | 14 (9%) | 25 (12%) | 39 (11%) | 0.28^2^ |
| Distant disease recurrence | 45 (29%) | 51 (25%) | 96 (27%) | 0.51^2^ |
| CRC-related mortality | 42 (27%) | 49 (24%) | 91 (25%) | 0.63^2^ |
| Overall mortality | 65 (41%) | 100 (50%) | 165 (46%) | 0.10^2^ |
| Follow-up time in months, median  (min – max) | 59.7 (3.4 – 148.6) | 57.1 (4.1 – 142.6) | 57.3 (3.4 – 148.6) | 0.50^1^ |

^1^Kruskal-Wallis rank sum test
^2^Pearson’s Chi-squared test
^3^Fisher’s exact test

**b. Characteristics of stage II patients included in the analysis of SLPI expression using the monoclonal antibody**

| **Clinicopathological variable** | **SLPI-low (n=95)** | **SLPI-high (n=117)** | **Total (n=212)** | ***P*-value** |
| --- | --- | --- | --- | --- |
| Age in years, median (min - max) | 71  (28 – 92) | 74  (40 – 92) | 73  (28 – 92) | 0.13^1^ |
| Gender  female  male | 42 (44%)  53 (56%) | 62 (53%)  55 (47%) | 104 (49%)  108 (51%) | 0.20^2^ |
| Tumor location  right (cecum until flexura lienalis)  left (flexura lienalis to rectum) | 45 (47%)  50 (53%) | 46 (39%)  71 (61%) | 91 (43%)  121 (57%) | 0.24^2^ |
| Tumor histological grade  well-differentiated  moderately differentiated  poorly differentiated | 7 (7%)  72 (76%)  16 (17%) | 9 (8%)  97 (83%)  11 (9%) | 16 (8%)  169 (80%)  27 (13%) | 0.27^2^ |
| Tumor maximal diameter in mm, median  (min - max) | 40  (20 – 130) | 40  (10 -100) | 40  (10 – 130) | 0.18^1^ |
| Tumor stage  T3  T4 | 83 (87%)  12 (13%) | 107 (92%)  10 (9%) | 190 (90%)  22 (10%) | 0.33^2^ |
| Mucinous differentiation | 17 (18%) | 27 (23%) | 44 (21%) | 0.36^2^ |
| MSI-status  MSI  MSS  Unknown | 13 (14%)  65 (68%)  17 (18%) | 21 (18%)  76 (65%)  20 (17%) | 34 (16%)  141 (67%)  37 (18%) | 0.41^2^ |
| Ulceration | 73 (77%) | 87 (74%) | 160 (76%) | 0.68^2^ |
| Angio-invasion | 14 (15%) | 8 (7%) | 22 (10%) | 0.06^2^ |
| Emergency surgery | 11 (12%) | 14 (12%) | 25 (12%) | 0.93^2^ |
| Perforation (pre-/per-/post-operative) | 7 (7%) | 13 (11%) | 20 (9%) | 0.35^2^ |
| Tumor spill | 3 (3%) | 3 (3%) | 6 (3%) | 1.00^3^ |
| Adjuvant chemotherapy | 17 (18%) | 16 (13%) | 33 (16%) | 0.40^2^ |
| Disease recurrence | 17 (18%) | 30 (26%) | 47 (22%) | 0.18^2^ |
| Local disease recurrence | 6 (6%) | 15 (13%) | 21 (10%) | 0.12^2^ |
| Distant disease recurrence | 16 (17%) | 21 (18%) | 37 (18%) | 0.83^2^ |
| CRC-related mortality | 15 (16%) | 20 (17%) | 35 (17%) | 0.80^2^ |
| Overall mortality | 31 (33%) | 53 (45%) | 84 (40%) | 0.06^2^ |
| Follow-up time in months, median  (min – max) | 73.8 (9.0 – 128.4) | 61.0 (5.3 – 139.6) | 63.5 (5.3 – 139.6) | 0.20^1^ |

^1^Kruskal-Wallis rank sum test
^2^Pearson’s Chi-squared test
^3^Fisher’s exact test

**c. Characteristics of stage III patients included in the analysis of SLPI expression using the monoclonal antibody**

| **Clinicopathological variable** | **SLPI-low (n=63)** | **SLPI-high (n=84)** | **Total (n=147)** | ***P*-value** |
| --- | --- | --- | --- | --- |
| Age in years, median (min - max) | 71  (34 – 87) | 73  (35 – 91) | 72  (34 – 91) | 0.99^1^ |
| Gender  female  male | 28 (44%)  35 (56%) | 37 (44%)  47 (56%) | 65 (44%)  82 (56%) | 0.96^2^ |
| Tumor location  right (cecum until flexura lienalis)  left (flexura lienalis to rectum) | 34 (54%)  29 (46%) | 33 (39%)  51 (61%) | 67 (46%)  80 (54%) | 0.08^2^ |
| Tumor histological grade  well-differentiated  moderately differentiated  poorly differentiated | 1 (2%)  47 (75%)  15 (24%) | 3 (4%)  67 (80%)  14 (17%) | 4 (3%)  114 (78%)  29 (20%) | 0.52^3^ |
| Tumor maximal diameter in mm, median  (min - max) | 40  (15 – 80) | 35  (10 – 100) | 40  (10 – 100) | 0.09^1^ |
| Tumor stage  T1  T2  T3  T4 | 2 (3%)  5 (8%)  51 (81%)  5 (8%) | 2 (2%)  14 (17%)  62 (74%)  6 (7%) | 4 (3%)  19 (13%)  113 (77%)  11 (8%) | 0.48^3^ |
| Nodal stage  N1  N2 | 40 (64%)  23 (37%) | 60 (71%)  24 (29%) | 100 (68%)  47 (32%) | 0.31^2^ |
| Mucinous differentiation | 10 (16%) | 21 (25%) | 31 (21%) | 0.18^2^ |
| MSI-status  MSI  MSS  Unknown | 10 (16%)  51 (81%)  2 (3%) | 12 (14%)  63 (75%)  9 (11%) | 22 (15%)  114 (78%)  11 (8%) | 0.95^2^ |
| Ulceration | 52 (83%) | 65 (77%) | 177 (80%) | 0.44^2^ |
| Angio-invasion | 20 (32%) | 28 (33%) | 48 (33%) | 0.84^2^ |
| Emergency surgery | 10 (16%) | 12 (14%) | 22 (15%) | 0.79^2^ |
| Perforation (pre-/per-/post-operative) | 5 (8%) | 6 (7%) | 11 (8%) | 1.00^3^ |
| Tumor spill | 1 (2%) | 5 (6%) | 6 (4%) | 0.24^3^ |
| Adjuvant chemotherapy | 35 (56%) | 48 (57%) | 83 (57%) | 0.85^2^ |
| Disease recurrence | 34 (54%) | 35 (42%) | 69 (47%) | 0.14^2^ |
| Local disease recurrence | 8 (13%) | 10 (12%) | 18 (12%) | 0.89^2^ |
| Distant disease recurrence | 29 (46%) | 30 (36%) | 59 (40%) | 0.21^2^ |
| CRC-related mortality | 27 (43%) | 29 (35%) | 56 (38%) | 0.30^2^ |
| Overall mortality | 34 (54%) | 47 (56%) | 81 (55%) | 0.81^2^ |
| Follow-up time in months, median  (min – max) | 45.7 (3.4 – 148.6) | 52.2 (4.1 – 142.6) | 50.4 (3.4 – 148.6) | 0.55^1^ |

^1^Kruskal-Wallis rank sum test
^2^Pearson’s Chi-squared test
^3^Fisher’s exact test

**d. Characteristics of stage III patients with MSS tumors included in the analysis of SLPI expression using the monoclonal antibody**

| **Clinicopathological variable** | **SLPI-low (n=51)** | **SLPI-high (n=63)** | **Total (n=114)** | ***P*-value** |
| --- | --- | --- | --- | --- |
| Age in years, median (min - max) | 71  (37 – 87) | 72  (38 – 91) | 72  (37 – 91) | 0.89^1^ |
| Gender  female  male | 18 (35%)  33 (65%) | 28 (44%)  35 (56%) | 46 (40%)  68 (60%) | 0.32^2^ |
| Tumor location  right (cecum until flexura lienalis)  left (flexura lienalis to rectum) | 24 (47%)  27 (53%) | 24 (38%)  39 (62%) | 48 (42%)  66 (58%) | 0.34^2^ |
| Tumor histological grade  well-differentiated  moderately differentiated  poorly differentiated | 1 (2%)  42 (82%)  8 (16%) | 3 (5%)  51 (81%)  9 (14%) | 4 (4%)  93 (82%)  17 (15%) | 0.85^3^ |
| Tumor maximal diameter in mm, median  (min - max) | 40  (15 – 80) | 30  (10 – 100) | 35  (10 – 100) | 0.19^1^ |
| Tumor stage  T1  T2  T3  T4 | 2 (4%)  5 (10%)  41 (80%)  3 (6%) | 2 (3%)  12 (19%)  44 (70%)  5 (8%) | 4 (4%)  17 (15%)  85 (75%)  8 (7%) | 0.49^3^ |
| Nodal stage  N1  N2 | 33 (65%)  18 (35%) | 46 (73%)  17 (27%) | 79 (69%)  35 (31%) | 0.34^2^ |
| Mucinous differentiation | 3 (6%) | 16 (25%) | 19 (17%) | **<0.01^2^** |
| Ulceration | 42 (82%) | 48 (76%) | 90 (79%) | 0.42^2^ |
| Angio-invasion | 17 (33%) | 20 (32%) | 37 (33%) | 0.86^2^ |
| Emergency surgery | 9 (18%) | 9 (14%) | 18 (16%) | 0.63^2^ |
| Perforation (pre-/per-/post-operative) | 5 (10%) | 5 (8%) | 10 (9%) | 0.75^3^ |
| Tumor spill | 1 (2%) | 4 (6%) | 5 (4%) | 0.38^3^ |
| Adjuvant chemotherapy | 30 (59%) | 36 (57%) | 66 (58%) | 0.86^2^ |
| Disease recurrence | 29 (57%) | 24 (38%) | 53 (47%) | 0.05^2^ |
| Local disease recurrence | 6 (12%) | 8 (13%) | 14 (12%) | 0.88^2^ |
| Distant disease recurrence | 25 (49%) | 20 (32%) | 45 (40%) | 0.06^2^ |
| CRC-related mortality | 23 (45%) | 22 (35%) | 45 (40%) | 0.27^2^ |
| Overall mortality | 30 (59%) | 37 (59%) | 67 (59%) | 0.99^2^ |
| Follow-up time in months, median  (min – max) | 44.9 (3.4 – 148.6) | 52.5 (4.1 – 142.6) | 46.9 (3.4 – 148.6) | 0.51^1^ |

^1^Kruskal-Wallis rank sum test
^2^Pearson’s Chi-squared test
^3^Fisher’s exact test

**e. Characteristics of stage III patients with MSS tumors who received adjuvant chemotherapy and were included in the analysis of SLPI expression using the monoclonal antibody**

| **Clinicopathological variable** | **SLPI-low (n=30)** | **SLPI-high (n=36)** | **Total (n=66)** | ***P*-value** |
| --- | --- | --- | --- | --- |
| Age in years, median (min - max) | 69 (37 – 83) | 66 (38 – 82) | 67  (37 – 83) | 0.79^1^ |
| Gender  female  male | 8 (27%)  22 (73%) | 13 (36%)  23 (64%) | 21 (32%)  45 (68%) | 0.41^2^ |
| Tumor location  right (cecum until flexura lienalis)  left (flexura lienalis to rectum) | 15 (50%)  15 (50%) | 14 (39%)  22 (61%) | 29 (44%)  37 (56%) | 0.37^2^ |
| Tumor histological grade  well-differentiated  moderately differentiated  poorly differentiated | 0 (0%)  25 (83%)  5 (17%) | 2 (6%)  28 (78%)  6 (17%) | 2 (3%)  53 (80%)  11 (17%) | 0.66^3^ |
| Tumor maximal diameter in mm, median  (min - max) | 35  (15 – 60) | 30  (10 – 90) | 33  (10 – 90) | 0.31^1^ |
| Tumor stage  T1  T2  T3  T4 | 0 (0%)  3 (10%)  25 (83%)  2 (7%) | 2 (6%)  6 (17%)  23 (64%)  5 (14%) | 2 (3%)  9 (14%)  48 (73%)  7 (11%) | 0.33^3^ |
| Nodal stage  N1  N2 | 16 (53%)  14 (47%) | 26 (72%)  10 (28%) | 42 (64%)  24 (36%) | 0.11^2^ |
| Mucinous differentiation | 2 (7%) | 4 (11%) | 6 (9%) | 0.68^3^ |
| Ulceration | 24 (80%) | 31 (86%) | 55 (83%) | 0.51^2^ |
| Angio-invasion | 12 (40%) | 12 (33%) | 24 (36%) | 0.58^2^ |
| Emergency surgery | 6 (20%) | 3 (8%) | 9 (14%) | 0.28^3^ |
| Perforation (pre-/per-/post-operative) | 2 (7%) | 1 (3%) | 3 (5%) | 0.59^3^ |
| Tumor spill | 1 (3%) | 1 (3%) | 2 (3%) | 1.00^3^ |
| Disease recurrence | 18 (60%) | 13 (36%) | 31 (47%) | 0.05^2^ |
| Local disease recurrence | 3 (10%) | 2 (6%) | 5 (3%) | 0.65^3^ |
| Distant disease recurrence | 17 (57%) | 12 (33%) | 29 (44%) | 0.06^2^ |
| CRC-related mortality | 14 (47%) | 11 (31%) | 25 (38%) | 0.18^2^ |
| Overall mortality | 17 (57%) | 15 (42%) | 32 (49%) | 0.23^2^ |
| Follow-up time in months, median  (min – max) | 45.1 (3.4 – 127.0) | 57.2 (4.1 – 127.4) | 53.0 (3.4 – 127.4) | 0.19^1^ |

^1^Kruskal-Wallis rank sum test
^2^Pearson’s Chi-squared test
^3^Fisher’s exact test

**Supplementary figure 2: Clinicopathological characteristics of CRC patients included in the analysis of SLPI expression.** Patients were classified as ‘SLPI-low’ or ‘SLPI-high’ based on the validated cut-offs. Characteristics of the patients included in the analysis of SLPI expression stained with the monoclonal antibody in the whole cohort (a), in stage II CRC patients (b), in stage III CRC patients (c), in stage III MSS CRC patients (d) and in stage III MSS CRC patients treated with adjuvant chemotherapy (e).

**Supplementary figure 3:**


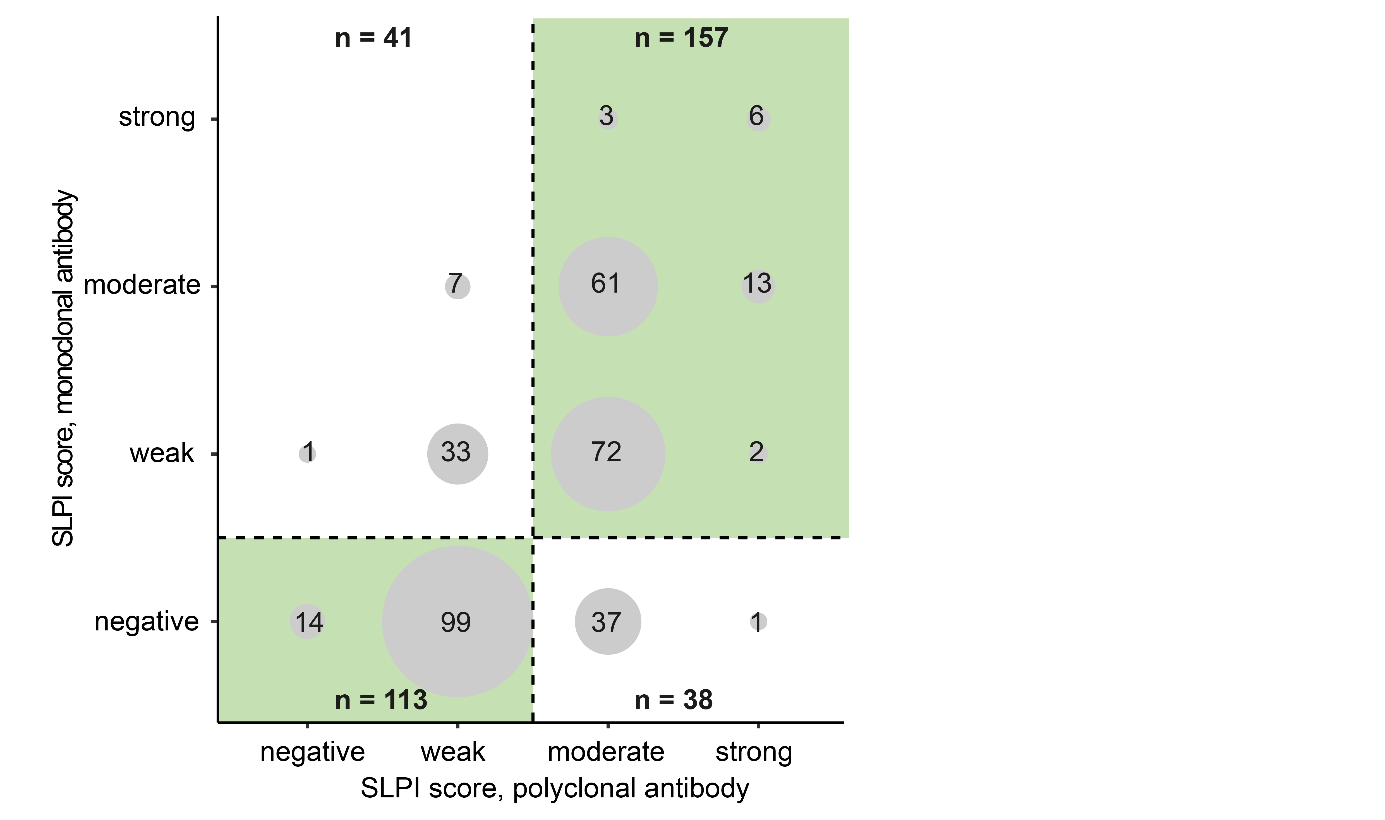


**Supplementary figure 3: Relationship between SLPI detected using the monoclonal antibody and SLPI detected using the polyclonal antibody**. SLPI expression was scored for both the monoclonal antibody staining and the polyclonal antibody staining in CRC tissues from 349 stage II or stage III patients. Green fields represent the cases in which dichotomization into the ‘SLPI-low’ or ‘SLPI-high’ group based on the validated cut-offs was not different for tissues stained using the monoclonal or polyclonal antibody (77% of patients). Dotted lines represent the cut-offs for the monoclonal and polyclonal antibody. The size of the circles represents the number of patients per group, which is also indicated in the circles.

**Supplementary figure 4:**


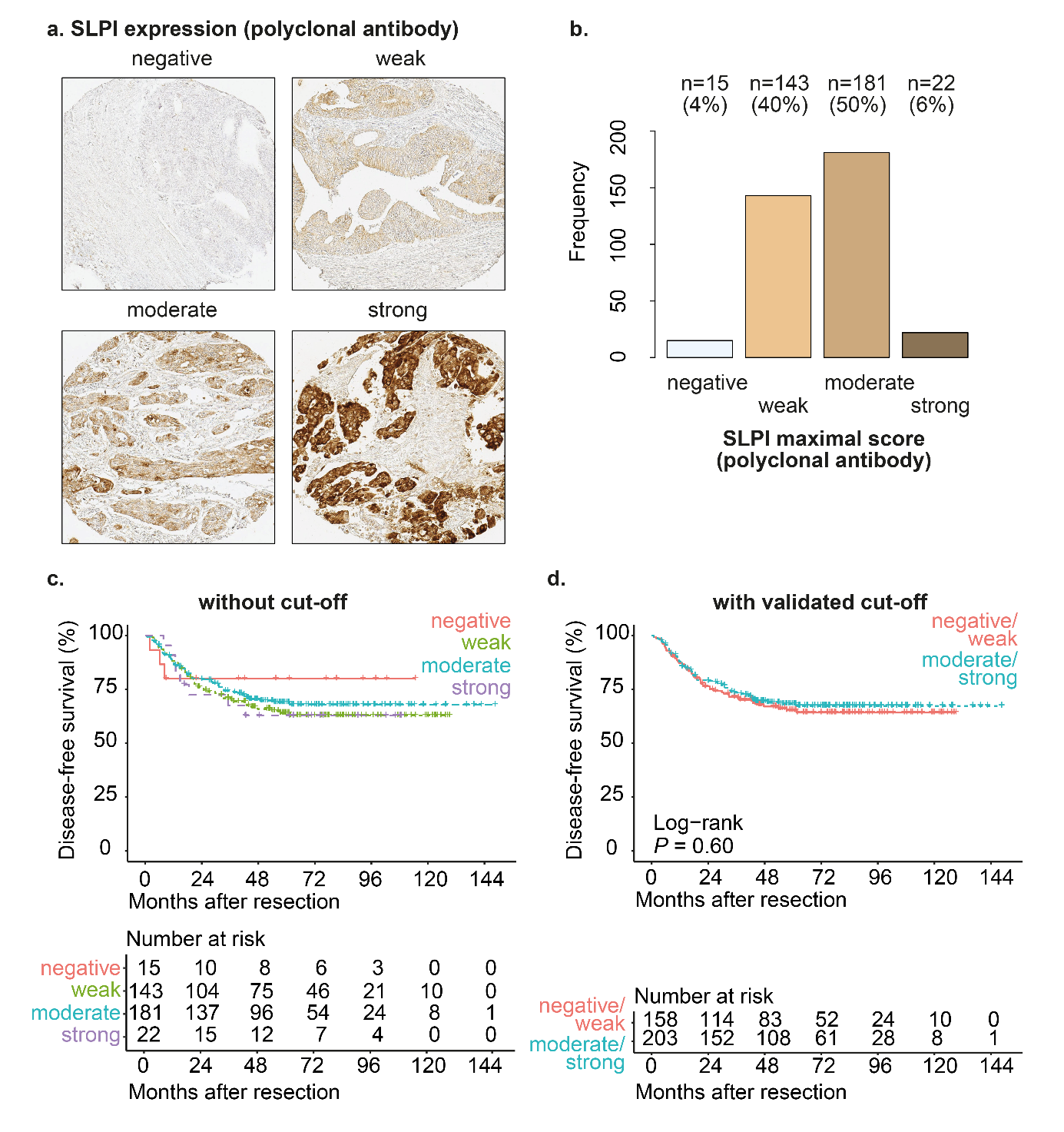


**Supplementary figure 4: SLPI expression detected with the polyclonal antibody in stage II and stage III CRC.** Examples of TMA cores of stage II or stage III CRC stained for SLPI using the polyclonal antibody (a). Frequencies and percentages of stage II or stage III CRC scored as ‘negative’, ‘weak’, ‘moderate’ or ‘strong’ after staining with the polyclonal SLPI antibody (b); only the maximal score for each patient was included. Kaplan-Meier curves for disease-free survival after resection of the primary tumor (in months) for the total study population of stage II and stage III CRC patients stratified by SLPI expression detected using the polyclonal antibody (c + d, HRR 0.91, *P*-value 0.60, 95% confidence interval 0.63 – 1.31). Curves without a cut-off (c) and with the validated cut-off (d) are shown. *P*-values were calculated using the log-rank test.

**Supplementary figure 5:**


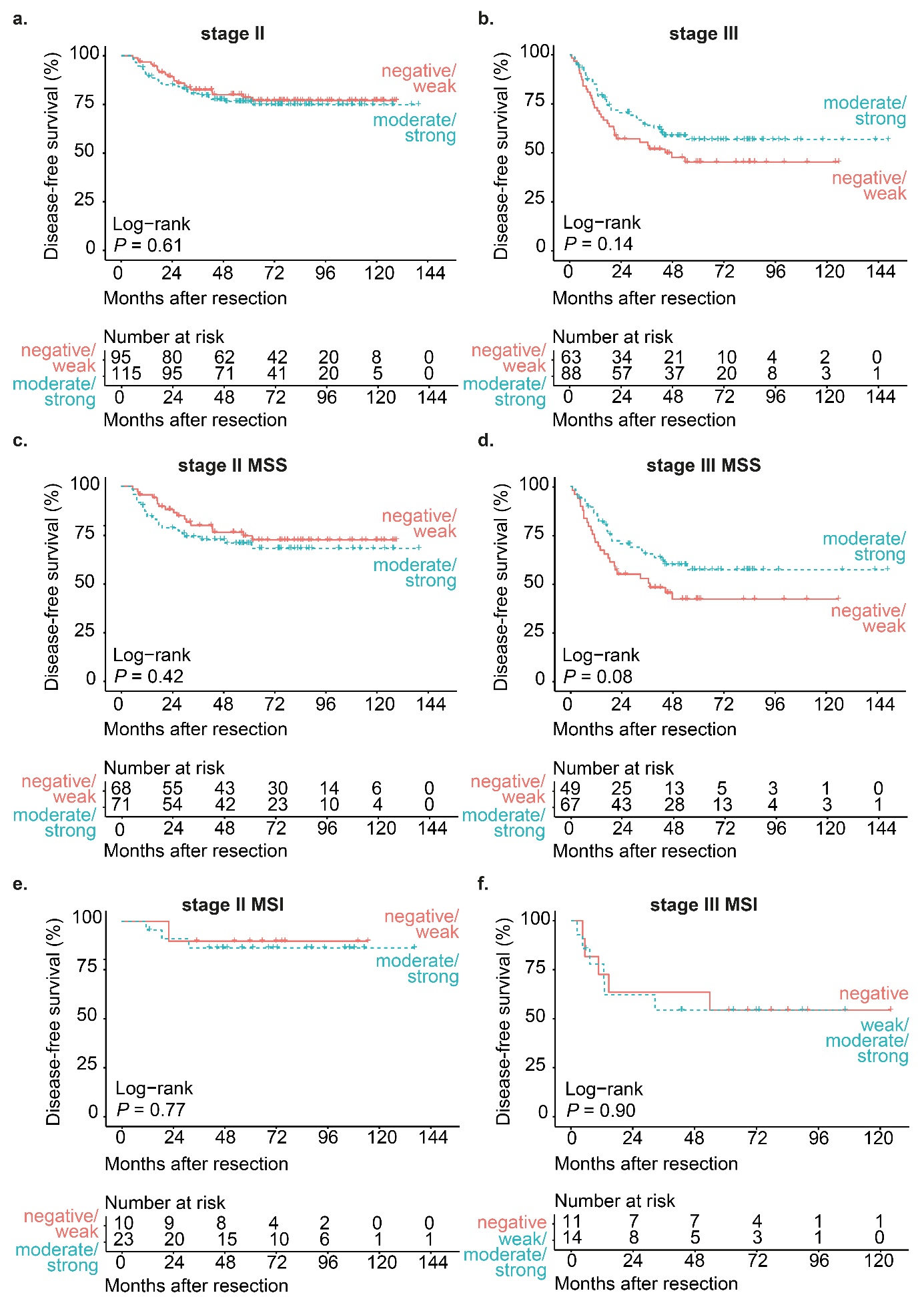


**Supplementary figure 5: SLPI expression detected with the polyclonal antibody in MSS and MSI CRC**. Kaplan-Meier curves for disease-free survival after resection of the primary tumor (in months) for either stage II CRC patients (a, HRR 1.16, *P*-value 0.61, 95% confidence interval 0.65 – 2.08) or stage III CRC patients (b, HRR 0.70, *P*-value 0.14, 95% confidence interval 0.44 – 1.13) stratified by SLPI expression detected using the polyclonal antibody. Kaplan-Meier curves for disease-free survival after resection of the primary tumor (in months) for stage II CRC patients with MSS (c, HRR 1.30, *P*-value 0.42, 95% confidence interval 0.69 – 2.47) or MSI tumors (e, HRR 1.39, *P*-value 0.77, 95% confidence interval 0.15 – 13.42) and stage III CRC patients with MSS (d, HRR 0.62, *P*-value 0.08, 95% confidence interval 0.36 – 1.06) or MSI tumors (f, HRR 1.08, *P*-value 0.90, 95% confidence interval 0.39 – 3.54) stratified by SLPI expression detected using the polyclonal antibody. Curves with the validated cut-off are shown. *P*-values were calculated using the log-rank test. MSS = micro-satellite stable. MSI = micro-satellite instable.

**Supplementary figure 6:**


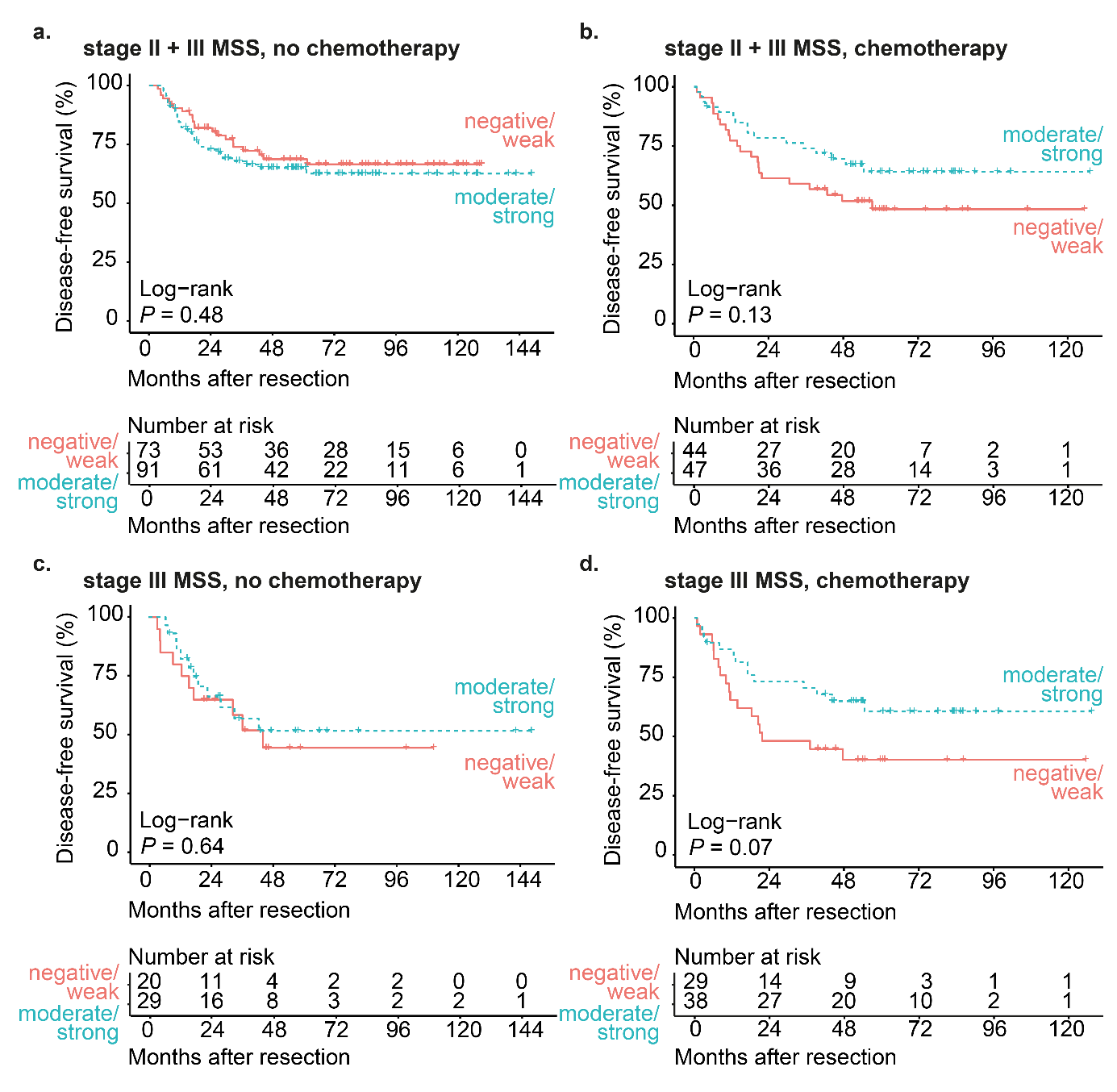


**Supplementary figure 6: SLPI expression detected with the polyclonal antibody in MSS CRC patients treated with adjuvant chemotherapy.** Kaplan-Meier curves for disease-free survival after resection of the primary tumor (in months) for stage II and stage III CRC patients with MSS tumors not treated with adjuvant chemotherapy (a, HRR 1.22, *P*-value 0.48, 95% confidence interval 0.70 – 2.10) or treated with adjuvant chemotherapy (b, HRR 1.63, *P*-value 0.13, 95% confidence interval 0.32 – 1.17) stratified by SLPI expression detected using the polyclonal antibody. Kaplan-Meier curves for disease-free survival after resection of the primary tumor (in months) for stage III CRC patients with MSS tumors not treated with adjuvant chemotherapy (c, HRR 0.82, *P*-value 0.64, 95% confidence interval 0.35 – 1.89) or treated with adjuvant chemotherapy (d, HRR 0.53, *P*-value 0.07, 95% confidence interval 0.26 – 1.07) stratified by SLPI expression detected using the polyclonal antibody. Curves with the validated cut-off are shown. *P*-values were calculated using the log-rank test. MSS = micro-satellite stable.

**Supplementary figure 7:**

**a. Characteristics of stage II and stage III patients included in the analysis of SLPI expression using the polyclonal antibody**

| **Clinicopathological variable** | **SLPI-low (n=158)** | **SLPI-high (n=203)** | **Total (n=361)** | ***P*-value** |
| --- | --- | --- | --- | --- |
| Age in years, median (min - max) | 72  (28 – 92) | 73  (36 – 93) | 73  (28 – 93) | 0.27^1^ |
| Gender  female  male | 76 (48%)  82 (52%) | 94 (46%)  109 (54%) | 170 (47%)  191 (53%) | 0.74^2^ |
| Tumor location  right (cecum until flexura lienalis)  left (flexura lienalis to rectum) | 71 (45%)  87 (55%) | 90 (44%)  113 (56%) | 161 (45%)  200 (55%) | 0.91^2^ |
| Tumor histological grade  well-differentiated  moderately differentiated  poorly differentiated | 7 (4%)  130 (82%)  21 (4%) | 16 (8%)  156 (77%)  31 (15%) | 23 (6%)  286 (79%)  52 (14%) | 0.33^2^ |
| Tumor maximal diameter in mm, median  (min - max) | 38  (10 - 130) | 40  (10 – 100) | 40  (10, 130) | 0.99^1^ |
| Stage  II III | 95 (60%)  63 (40%) | 115 (57%)  88 (43%) | 210 (58%)  151 (42%) | 0.51^2^ |
| Tumor stage  T1  T2  T3  T4 | 1 (1%)  4 (3%)  136 (86%)  17 (11%) | 3 (2%)  15 (7%)  166 (82%)  19 (9%) | 4 (1%)  19 (5%)  302 (84%)  36 (10%) | 0.18^3^ |
| Nodal stage (Stage III patients only)  N1  N2 | 42 (67%)  21 (33%) | 63 (72%)  25 (28%) | 105 (70%)  46 (31%) | 0.52^2^ |
| Mucinous differentiation | 25 (16%) | 49 (24%) | 74 (21%) | 0.05^2^ |
| MSI-status  MSI  MSS  Unknown | 21 (13%)  117 (74%)  20 (13%) | 37 (18%)  138 (68%)  28 (14%) | 58 (16%)  255 (71%)  48 (13%) | 0.39^2^ |
| Ulceration | 124 (79%) | 151 (74%) | 275 (76%) | 0.37^2^ |
| Angio-invasion | 31 (20%) | 36 (18%) | 67 (19%) | 0.65^2^ |
| Emergency surgery | 23 (15%) | 26 (13%) | 49 (13.6%) | 0.63^2^ |
| Perforation (pre-/per-/post-operative) | 16 (10%) | 15 (7%) | 31 (9%) | 0.36^2^ |
| Tumor spill | 3 (2%) | 9 (4%) | 12 (3%) | 0.18^2^ |
| Adjuvant chemotherapy | 56 (35%) | 63 (31%) | 119 (33%) | 0.38^2^ |
| Disease recurrence | 53 (34%) | 62 (31%) | 115 (32%) | 0.54^2^ |
| Local disease recurrence | 18 (11%) | 21 (10%) | 39 (11%) | 0.75^2^ |
| Distant disease recurrence | 43 (27%) | 51 (25%) | 94 (26%) | 0.65^2^ |
| CRC-related mortality | 40 (25%) | 52 (26%) | 92 (26%) | 0.95^2^ |
| Overall mortality | 64 (41%) | 100 (49%) | 164 (45%) | 0.10^2^ |
| Follow-up time in months, median  (min – max) | 60.1 (4.3 – 129.2) | 57.1 (3.4 – 148.6) | 57.4 (3.4 – 148.6) | 0.50^1^ |

^1^Kruskal-Wallis rank sum test
^2^Pearson’s Chi-squared test
^3^Fisher’s exact test

**b. Characteristics of stage II patients included in the analysis of SLPI expression using the polyclonal antibody**

| **Clinicopathological variable** | **SLPI-low (n=95)** | **SLPI-high (n=115)** | **Total (n=210)** | ***P*-value** |
| --- | --- | --- | --- | --- |
| Age in years, median (min - max) | 73  (28 – 92) | 73  (40 – 92) | 73  (28 – 92) | 0.29^1^ |
| Gender  female  male | 54 (47%)  61 (53%) | 50 (53%)  45 (47%) | 104 (50%)  106 (51%) | 0.41^2^ |
| Tumor location  right (cecum until flexura lienalis)  left (flexura lienalis to rectum) | 38 (40%)  57 (60%) | 53 (46%)  62 (54%) | 91 (43%)  119 (57%) | 0.38^2^ |
| Tumor histological grade  well-differentiated  moderately differentiated  poorly differentiated | 6 (6%)  80 (84%)  9 (10%) | 11 (10%)  90 (78%)  14 (12%) | 17 (8%)  170 (81%)  23 (11%) | 0.54^2^ |
| Tumor maximal diameter in mm, median  (min - max) | 35  (12 – 130) | 40  (10 -100) | 40  (10 – 130) | 0.61^1^ |
| Tumor stage  T3  T4 | 84 (88%)  11 (12%) | 103 (90%)  12 (10%) | 187 (89%)  23 (11%) | 0.79^2^ |
| Mucinous differentiation | 15 (16%) | 29 (25%) | 44 (21%) | 0.10^2^ |
| MSI-status  MSI  MSS  Unknown | 10 (11%)  68 (72%)  17 (18%) | 23 (20%)  71 (62%)  21 (18%) | 33 (16%)  139 (66%)  38 (18%) | 0.05^2^ |
| Ulceration | 69 (73%) | 87 (76%) | 156 (74%) | 0.62^2^ |
| Angio-invasion | 12 (13%) | 8 (7%) | 20 (10%) | 0.16^2^ |
| Emergency surgery | 11 (12%) | 14 (12%) | 25 (12%) | 0.90^2^ |
| Perforation (pre-/per-/post-operative) | 9 (10%) | 11 (10%) | 20 (10%) | 0.98^2^ |
| Tumor spill | 2 (2%) | 4 (4%) | 6 (3%) | 0.70^3^ |
| Adjuvant chemotherapy | 18 (19%) | 15 (13%) | 33 (16%) | 0.24^2^ |
| Disease recurrence | 20 (21%) | 27 (24%) | 47 (22%) | 0.68^2^ |
| Local disease recurrence | 9 (10%) | 11 (10%) | 20 (10%) | 0.98^2^ |
| Distant disease recurrence | 15 (16%) | 22 (19%) | 37 (18%) | 0.53^2^ |
| CRC-related mortality | 13 (14%) | 22 (19%) | 35 (17%) | 0.29^2^ |
| Overall mortality | 31 (33%) | 52 (45%) | 83 (40%) | 0.06^2^ |
| Follow-up time in months, median  (min – max) | 71.9 (8.9 – 129.2) | 59.0 (5.3 – 139.6) | 64.3 (5.3 – 139.6) | 0.20^1^ |

^1^Kruskal-Wallis rank sum test
^2^Pearson’s Chi-squared test
^3^Fisher’s exact test

**c. Characteristics of stage III patients included in the analysis of SLPI expression using the polyclonal antibody**

| **Clinicopathological variable** | **SLPI-low (n=63)** | **SLPI-high (n=88)** | **Total (n=151)** | ***P*-value** |
| --- | --- | --- | --- | --- |
| Age in years, median (min - max) | 71  (34 – 87) | 73  (36 – 93) | 72  (34 – 93) | 0.64^1^ |
| Gender  female  male | 26 (41%)  37 (59%) | 40 (46%)  48 (55%) | 66 (44%)  85 (56%) | 0.61^2^ |
| Tumor location  right (cecum until flexura lienalis)  left (flexura lienalis to rectum) | 33 (52%)  30 (48%) | 37 (42%)  51 (58%) | 70 (46%)  81 (54%) | 0.21^2^ |
| Tumor histological grade  well-differentiated  moderately differentiated  poorly differentiated | 1 (2%)  50 (79%)  12 (19%) | 5 (6%)  66 (75%)  17 (19%) | 6 (4%)  116 (77%)  29 (19%) | 0.58^3^ |
| Tumor maximal diameter in mm, median  (min - max) | 40  (10 – 70) | 35  (12 – 100) | 35  (10 – 100) | 0.63^1^ |
| Tumor stage  T1  T2  T3  T4 | 1 (2%)  4 (6%)  52 (83%)  6 (10%) | 3 (3%)  15 (17%)  63 (72%)  7 (8%) | 4 (3%)  19 (13%)  115 (76%)  13 (9%) | 0.21^3^ |
| Nodal stage  N1  N2 | 42 (67%)  21 (33%) | 63 (72%)  25 (28%) | 105 (70%)  46 (31%) | 0.52^2^ |
| Mucinous differentiation | 10 (16%) | 20 (23%) | 30 (20%) | 0.30^2^ |
| MSI-status  MSI  MSS  Unknown | 11 (18%)  49 (78%)  3 (5%) | 14 (16%)  67 (76%)  7 (8%) | 25 (17%)  116 (77%)  10 (7%) | 0.87^2^ |
| Ulceration | 55 (87%) | 64 (73%) | 119 (80%) | **0.03^2^** |
| Angio-invasion | 19 (30%) | 28 (32%) | 47 (31%) | 0.83^2^ |
| Emergency surgery | 12 (19%) | 12 (14%) | 24 (16%) | 0.37^2^ |
| Perforation (pre-/per-/post-operative) | 7 (11%) | 4 (5%) | 11 (7%) | 0.20^3^ |
| Tumor spill | 1 (2%) | 5 (6%) | 6 (4%) | 0.40^3^ |
| Adjuvant chemotherapy | 38 (60%) | 48 (55%) | 86 (57%) | 0.48^2^ |
| Disease recurrence | 33 (52%) | 35 (40%) | 68 (45%) | 0.13^2^ |
| Local disease recurrence | 9 (14%) | 10 (11%) | 19 (13%) | 0.59^2^ |
| Distant disease recurrence | 28 (44%) | 29 (33%) | 57 (38%) | 0.15^2^ |
| CRC-related mortality | 27 (43%) | 30 (34%) | 57 (38%) | 0.27^2^ |
| Overall mortality | 33 (52%) | 48 (55%) | 81 (54%) | 0.79^2^ |
| Follow-up time in months, median  (min – max) | 46.4 (4.3 – 127.0) | 51.5 (3.4 – 148.6) | 50.4 (3.4 – 148.6) | 0.62^1^ |

^1^Kruskal-Wallis rank sum test
^2^Pearson’s Chi-squared test
^3^Fisher’s exact test

**d. Characteristics of stage III patients with MSS tumors included in the analysis of SLPI expression using the polyclonal antibody**

| **Clinicopathological variable** | **SLPI-low (n=49)** | **SLPI-high (n=67)** | **Total (n=116)** | ***P*-value** |
| --- | --- | --- | --- | --- |
| Age in years, median (min - max) | 72  (37 – 87) | 72  (36 – 91) | 72  (36 – 91) | 0.91^1^ |
| Gender  female  male | 18 (37%)  31 (63%) | 29 (43%)  38 (57%) | 47 (41%)  69 (60%) | 0.48^2^ |
| Tumor location  right (cecum until flexura lienalis)  left (flexura lienalis to rectum) | 22 (45%)  27 (55%) | 27 (40%)  40 (60%) | 49 (42%)  67 (58%) | 0.62^2^ |
| Tumor histological grade  well-differentiated  moderately differentiated  poorly differentiated | 1 (2%)  40 (82%)  8 (16%) | 5 (8%)  52 (78%)  10 (15%) | 6 (5%)  92 (79%)  18 (16%) | 0.58^3^ |
| Tumor maximal diameter in mm, median  (min - max) | 40  (10 – 70) | 30  (12 – 100) | 33  (10 – 100) | 0.24^1^ |
| Tumor stage  T1  T2  T3  T4 | 1 (2%)  4 (8%)  39 (80%)  5 (10%) | 3 (5%)  13 (19%)  46 (69%)  5 (8%) | 4 (3%)  17 (15%)  85 (73%)  10 (9%) | 0.31^3^ |
| Nodal stage  N1  N2 | 32 (65%)  17 (35%) | 48 (72%)  19 (28%) | 80 (69%)  36 (31%) | 0.47^2^ |
| Mucinous differentiation | 5 (10%) | 15 (22%) | 20 (17%) | 0.09^2^ |
| Ulceration | 42 (86%) | 48 (72%) | 90 (78%) | 0.07^2^ |
| Angio-invasion | 15 (31%) | 23 (34%) | 38 (33%) | 0.67^2^ |
| Emergency surgery | 10 (20%) | 9 (13%) | 19 (16%) | 0.32^2^ |
| Perforation (pre-/per-/post-operative) | 7 (14%) | 3 (5%) | 10 (9%) | 0.09^2^ |
| Tumor spill | 1 (2%) | 4 (6%) | 5 (4%) | 0.40^2^ |
| Adjuvant chemotherapy | 29 (59%) | 38 (57%) | 67 (58%) | 0.79^2^ |
| Disease recurrence | 27 (55%) | 26 (39%) | 53 (46%) | 0.08^2^ |
| Local disease recurrence | 6 (12%) | 9 (13%) | 15 (13%) | 0.85^2^ |
| Distant disease recurrence | 24 (49%) | 20 (30%) | 44 (38%) | **0.04^2^** |
| CRC-related mortality | 23 (47%) | 23 (34%) | 46 (40%) | 0.17^2^ |
| Overall mortality | 29 (59%) | 39 (58%) | 68 (59%) | 0.92^2^ |
| Follow-up time in months, median  (min – max) | 45.3 (4.3 – 127.0) | 51.9 (3.4 – 148.6) | 46.9 (3.4 – 148.6) | 0.36^1^ |

^1^Kruskal-Wallis rank sum test
^2^Pearson’s Chi-squared test
^3^Fisher’s exact test

**e. Characteristics of stage III patients with MSS tumors who received adjuvant chemotherapy and were included in the analysis of SLPI expression using the polyclonal antibody**

| **Clinicopathological variable** | **SLPI-low (n=29)** | **SLPI-high (n=38)** | **Total (n=67)** | ***P*-value** |
| --- | --- | --- | --- | --- |
| Age in years, median (min - max) | 66  (37 – 83) | 67 (36 – 82) | 66  (36 – 83) | 0.80^1^ |
| Gender  female  male | 10 (35%)  19 (66%) | 12 (32%)  26 (68%) | 22 (33%)  45 (67%) | 0.80^2^ |
| Tumor location  right (cecum until flexura lienalis)  left (flexura lienalis to rectum) | 15 (52%)  14 (48%) | 15 (40%)  23 (61%) | 30 (45%)  37 (55%) | 0.32^2^ |
| Tumor histological grade  well-differentiated  moderately differentiated  poorly differentiated | 0 (0%)  25 (86%)  4 (14%) | 4 (11%)  27 (71%)  7 (18%) | 4 (6%)  52 (78%)  11 (16%) | 0.18^3^ |
| Tumor maximal diameter in mm, median  (min - max) | 35  (10 – 60) | 30  (12 – 60) | 30  (10 – 60) | 0.40^1^ |
| Tumor stage  T1  T2  T3  T4 | 0 (0%)  2 (7%)  24 (83%)  3 (10%) | 2 (5%)  7 (18%)  24 (63%)  5 (13%) | 2 (3%)  9 (13%)  48 (72%)  8 (12%) | 0.30^3^ |
| Nodal stage  N1  N2 | 17 (59%)  12 (41%) | 25 (86%)  13 (34%) | 42 (63%)  25 (37%) | 0.55^2^ |
| Mucinous differentiation | 2 (7%) | 4 (11%) | 6 (9%) | 0.69^3^ |
| Ulceration | 24 (83%) | 30 (79%) | 54 (81%) | 0.70^2^ |
| Angio-invasion | 10 (35%) | 14 (37%) | 24 (36%) | 0.84^2^ |
| Emergency surgery | 6 (21%) | 4 (11%) | 10 (15%) | 0.31^3^ |
| Perforation (pre-/per-/post-operative) | 3 (10%) | 0 (0%) | 3 (5%) | 0.08^3^ |
| Tumor spill | 1 (3%) | 1 (3%) | 2 (3%) | 1.00^3^ |
| Disease recurrence | 17 (59%) | 14 (37%) | 31 (46%) | 0.09^3^ |
| Local disease recurrence | 3 (10%) | 3 (8%) | 6 (9%) | 1.00^3^ |
| Distant disease recurrence | 17 (59%) | 11 (29%) | 28 (42%) | **0.02^2^** |
| CRC-related mortality | 14 (48%) | 12 (32%) | 26 (39%) | 0.17^2^ |
| Overall mortality | 16 (55%) | 17 (45%) | 33 (49%) | 0.40^2^ |
| Follow-up time in months, median  (min – max) | 46.4 (4.3 – 127.0) | 56.0 (3.4 – 127.4) | 52.5 (3.4 – 127.4) | 0.25^1^ |

^1^Kruskal-Wallis rank sum test
^2^Pearson’s Chi-squared test
^3^Fisher’s exact test

**Supplementary figure 7: Clinicopathological characteristics of CRC patients included in the analysis of SLPI expression.** Patients were classified as ‘SLPI-low’ or ‘SLPI-high’ based on the validated cut-offs. Characteristics of the patients included in the analysis of SLPI expression stained with the polyclonal antibody in the whole cohort (a), in stage II CRC patients (b), in stage III CRC patients (c), in stage III MSS CRC patients (d) and in stage III MSS CRC patients treated with adjuvant chemotherapy (e).
